# Association between serum total bilirubin levels and 28-day all-cause mortality after intracerebral hemorrhage

**DOI:** 10.3389/fneur.2025.1529415

**Published:** 2025-02-12

**Authors:** Dachang Qiu, Guangwei Li, Yongfei Dong

**Affiliations:** ^1^Wuxi School of Medicine, Jiangnan University, Wuxi, Jiangsu, China; ^2^Institute of Artificial Intelligence, Hefei Comprehensive National Science Center, Hefei, Anhui, China; ^3^Department of Neurosurgery, The First Affiliated Hospital of USTC, Division of Life Sciences and Medicine, University of Science and Technology of China, Hefei, Anhui, China

**Keywords:** total serum bilirubin, intracerebral hemorrhage, 28 days all-cause mortality, MIMIC-IV database, prognosis

## Abstract

**Background:**

Intracerebral hemorrhage (ICH) is associated with high mortality and morbidity rates. Although some studies have indicated a correlation between serum bilirubin levels and ICH severity, evidence of the relationship between serum total bilirubin (TBIL) and ICH outcomes remains lacking.

**Methods:**

A total of 914 patients from the Medical Information Mart for Intensive Care IV database met the eligibility criteria and were included in the study. The patients were categorized into two groups based on whether they survived for 28 days following admission to hospital. The association between serum TBIL levels and 28-day survival in patients with ICH was investigated using Spearman’s correlation analysis and restricted cubic splines. The effect of serum TBIL levels on survival time and rate in the 28-day period was analyzed using Kaplan–Meier curves and restricted mean survival times. Univariate Cox regression, least absolute shrinkage and selection operator regression, and multivariate Cox regression were used to identify risk factors associated with 28-day all-cause mortality. Finally, subgroup analysis was performed to verify the stability of the association between serum TBIL levels and 28-day all-cause mortality in patients with ICH.

**Results:**

A negative relationship was revealed between TBIL levels and survival (*p* < 0.001, correlation = −0.174). Restricted cubic spline analysis revealed a nonlinear link between mean serum TBIL levels and 28-day all-cause mortality (p for nonlinear = 0.001). Patients with ICH and higher serum TBIL levels had significantly reduced survival times and rates compared with those with lower serum TBIL levels (*p* < 0.001). Serum TBIL level was identified as a significant risk factor for 28-day all-cause mortality in patients with ICH (hazard ratio [95% confidence interval] = 1.121 [1.063–1.182], *p* < 0.001). Subgroup analyses revealed that the assessed variables had no influence on the association between serum TBIL levels and 28-day all-cause mortality.

**Conclusion:**

Higher serum TBIL levels are associated with a greater risk of mortality within 28 days in patients with ICH, whereas lower serum TBIL levels are associated with prolonged survival.

## Introduction

Intracerebral hemorrhage (ICH), which is caused by the rupture of blood vessels in the brain, leads to blood accumulation within the brain parenchyma (1, 2). The resultant hematoma creates mechanical damage, and inflammation triggered by blood degradation products can rapidly induce neuronal cell death, brain swelling, white matter injury, and compromise of the blood–brain barrier, all of which contribute to the elevated rates of mortality and disability associated with ICH (3–6). ICH accounts for approximately 27% of all newly reported stroke incidents, with an annual global incidence of 42 cases per 100,000 population (7). In 2019, ICH was the second most common cause of death worldwide and the third most common cause of death or disability (8). As the global population ages and use of antiplatelet therapies increases, the incidence of ICH also increases (7), posing a major challenge for healthcare systems worldwide.

Bilirubin, a product of hemoglobin degradation, is frequently used in clinical practice as a biomarker of hepatic diseases (9). Recent studies have indicated its importance in neurological conditions. It has been reported that bilirubin worsens brain injury in mouse models of cerebral infarction via the TRPM2 pathway (10). Moreover, a notable association has been demonstrated between serum bilirubin levels and mortality rates in individuals with traumatic brain injury (11). Another study found a linear correlation between serum bilirubin levels and the severity of ischemic stroke (p for nonlinearity = 0.55) (12). In patients with ICH, a positive correlation was observed between serum bilirubin levels and ICH severity at presentation. High bilirubin levels were associated with poorer Glasgow Coma Scale scores on admission (rs = −0.17, *p* = 0.011). The levels of total bilirubin (TBIL), direct bilirubin, and albumin were all significantly related to discharge outcomes (*p* = 0.036, *p* = 0.014, and *p* = 0.016, respectively) (13). Several studies have indicated that bilirubin and its oxidized forms may increase the severity of cerebral hemorrhage (14–16). Additionally, it has been proposed that serum bilirubin levels may predict hematoma growth after ICH (17). Despite indications that bilirubin levels are related to ICH severity, the underlying mechanisms remain unclear, particularly regarding the association between serum bilirubin levels and mortality due to ICH.

This retrospective study therefore investigated the association between serum bilirubin levels and ICH-associated mortality. Our goal was to increase understanding of this association and further clarify the relationship between serum bilirubin levels and the outcomes and prognosis of ICH.

## Materials and methods

### Data access

The information used in this study was obtained from the Medical Information Mart for Intensive Care IV (MIMIC-IV) database version 3.1. This extensive dataset includes the de-identified records of patients admitted to either the emergency department or intensive care unit (ICU) of the Beth Israel Deaconess Medical Center (Boston, MA, USA) between 2008 and 2022 (18). The collection of patient information and establishment of the database were reviewed by the Institutional Review Board of the Beth Israel Deaconess Medical Center, which waived the requirement for informed consent and approved the data-sharing initiative; no additional ethical approval was required for the present study (18, 19). Dachang Qiu, the primary author of this study, completed the requisite training and examinations mandated by the US National Institutes of Health and was given authorized access to the MIMIC-IV database for the purposes of data extraction (record ID: 65770488).

### Eligibility criteria

Researchers with significant expertise in clinical studies defined the inclusion and exclusion criteria for the study. Adult patients with a primary diagnosis of non-traumatic ICH in the ICU were included. The exclusion criteria were as follows: (1) repeated admittance to hospital or ICU, (2) died within 24 h of admission, (3) fewer than two serum bilirubin level tests performed after admission, and (4) essential clinical information missing.

### Core variables and outcomes

The primary variable in this study was the average serum TBIL level. The research endpoint was defined as mortality within 28 days of admission. The 28-day all-cause mortality rate was defined as the proportion of total deaths attributable to any cause within a 28-day hospital admission compared to the average population of that demographic during the equivalent timeframe.

### Clinical data collection

Clinical data on the following were collected from the database: (1) demographic factors such as age, sex, and ethnicity; (2) comorbidities such as hypertension, type 2 diabetes, hyperlipidemia, cancer, heart failure, ischemic heart disease, pneumonia, chronic bronchitis, kidney disease, and hepatic disorders; (3) physical assessments of respiration rate, heart rate, systolic average blood pressure, diastolic average blood pressure, and body weight; (4) laboratory analysis of average TBIL, initial TBIL, blood potassium, aspartate transaminase, alanine transaminase, albumin, creatinine, lactate dehydrogenase, blood glucose, triglyceride, serum sodium, and total serum calcium levels, hematocrit, leukocyte count, erythrocyte count, platelet count, and hemoglobin levels; and (5) disease severity measured using the Glasgow Coma Scale, Simplified Acute Physiology Score II, and Oxford Acute Severity of Illness Score.

### Statistical analysis

DecisionLinnc. software version 1.0. was used to conduct statistical analyses (20). This platform merges various programming language environments and streamlines data handling, analysis, and machine-learning processes via a graphical interface. Variables with more than 25% of values missing were discarded. Multiple imputation was used to fill in missing data if between 5 and 25% of values were missing. Mean imputation was applied if <5% of values were missing. The normality of the data was analyzed after sorting; the results are shown in Supplementary Table S1. Continuous variables displaying skewed distributions are described as median (Q1 – Q3); those with normal distributions are expressed as mean ± standard deviation. Categorical variables are reported as number and frequency. An independent samples t-test was used to compare the differences between two groups of normally distributed continuous variables. The Wilcoxon rank-sum test was applied to compare two groups of non-normally distributed continuous variables, whereas the chi-square test was used to compare two groups of categorical variables. Spearman’s correlation analysis was performed to evaluate the relationship between serum TBIL levels and patient survival. Restricted cubic splines were used to assess the nonlinear association between serum bilirubin levels and 28-day all-cause mortality. The participants were divided into two groups based on median TBIL level, and Kaplan–Meier survival analysis was performed to investigate differences in survival rates between the two groups. Univariate Cox regression analysis was conducted to identify risk factors associated with 28-day mortality. Least absolute shrinkage and selection operator (LASSO) regression was then performed on the identified risk factors to determine the variables with the greatest influence. Multivariable Cox regression was used to examine the association between serum bilirubin levels and 28-day mortality. Furthermore, subgroup analyses were conducted according to predetermined group classifications, and tests for interactions were performed. Statistical significance was defined as a two-tailed *p* < 0.05.

## Results

### Patient selection and clinical characteristics

**Figure 1:**
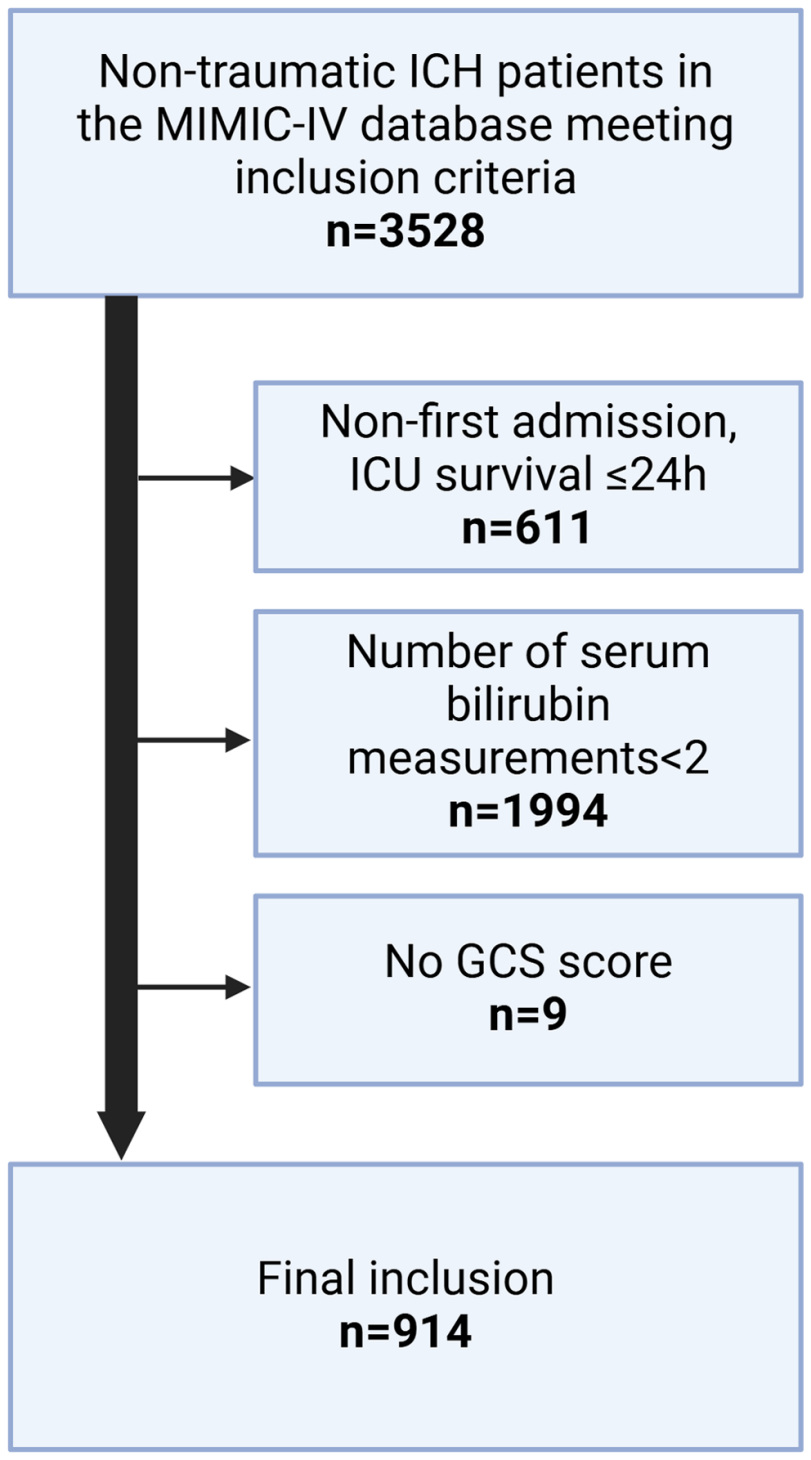
Process flowchart for inclusion/exclusion of study.

The patient selection process is depicted in Figure 1. A total of 914 participants were finally included in the analysis. Patients were divided into two groups based on whether death occurred within 28 days of admission. Of the 914 patients, 698 survived for 28 days after admission and 216 died during this period. The median ages of the deceased and survivor groups were 70 and 67 years, respectively (*p* < 0.001). Significant differences were also observed between the two groups in terms of the mean systolic blood pressure (*p* < 0.001) and average diastolic blood pressure (*p* < 0.001). The average serum TBIL level in the survivor group was 0.54 (0.36–0.83) mg/dL, which was significantly lower than that of the deceased group, at 0.70 (0.45–1.33) mg/dL (*p* < 0.001). The overall median serum TBIL level in all patients was 0.55 mg/dL. Patient characteristics are summarized in Table 1.

**Table 1:** Characteristics of the participants.

Variable	Overall	Death within 28 days		*p*-value
		No	Yes	
	*N* = 914	*N* = 698	*N* = 216	
Age	67.00 (57.00–78.00)	66.00 (55.00–77.00)	70.00 (61.00–82.50)	<0.001
RR, time/min	18.50 (16.00–22.00)	18.00 (15.00–22.00)	19.00 (16.00–23.00)	0.007
HR, time/min	84.00 (72.00–97.00)	84.00 (73.00–95.00)	86.00 (72.00–100.00)	0.107
Sabp, mmHg	137.00 (119.00–154.00)	139.00 (122.00–157.00)	131.00 (107.00–145.00)	0.001
Dabp, mmHg	66.50 (55.00–76.00)	67.00 (57.50–77.00)	60.50 (52.00–73.00)	0.003
Weight, Kg	76.75 (64.70–92.00)	77.00 (65.20–93.30)	75.20 (62.55–88.20)	0.111
GCS	14.00 (11.00–15.00)	14.00 (11.00–15.00)	13.00 (8.50–15.00)	0.082
Sapsii	34.00 (27.00–43.00)	31.00 (26.00–39.00)	42.00 (35.00–51.00)	<0.001
Oasis	32.00 (27.00–38.00)	31.00 (26.00–36.00)	37.00 (32.00–43.00)	<0.001
Average serum total bilirubin, mg/dL	0.55 (0.40–0.90)	0.54 (0.36–0.83)	0.70 (0.45–1.33)	<0.001
First serum total bilirubin, mg/dL	0.60 (0.40–1.00)	0.60 (0.40–1.00)	0.70 (0.40–1.20)	0.006
Blood potassium, mEq/L	3.90 (3.60–4.30)	3.90 (3.60–4.30)	4.00 (3.60–4.50)	0.018
AST, IU/L	33.00 (21.00–58.00)	31.50 (20.00–56.00)	37.50 (24.00–71.50)	<0.001
ALT, IU/L	24.50 (16.00–47.00)	25.00 (16.00–47.00)	24.00 (16.00–43.00)	0.716
Albumin, g/dL	3.40 (3.00–3.70)	3.40 (3.10–3.80)	3.30 (2.90–3.70)	0.002
Creatinine, mg/dL	0.90 (0.70–1.20)	0.90 (0.70–1.10)	1.00 (0.70–1.45)	<0.001
Lactate dehydrogenase, IU/L	265.00 (202.00–348.00)	254.00 (199.00–333.00)	290.00 (229.50–414.50)	<0.001
Blood glucose, mg/dL	129.00 (108.00–162.00)	126.00 (106.00–158.00)	140.50 (116.50–177.00)	<0.001
Triglycerides, mg/dL	110.00 (79.00–153.00)	110.00 (79.00–147.00)	110.00 (74.00–177.50)	0.983
PTT, second	28.55 (18.9–150)	28.4 (19–150)	29.1 (18.9–150)	0.061
PT, second	13 (9.9–65.8)	12.8 (9.9–65.8)	13.1 (10.1–64.6)	0.324
INR	1.2 (0.9–5.8)	1.2 (0.9–5.8)	1.2 (1–5.6)	0.082
Blood Sodium, mEq/L	139.00 (137.00–142.00)	139.00 (137.00–142.00)	140.00 (136.50–143.00)	0.391
Serum total calcium, mg/dL	8.70 (8.20–9.10)	8.70 (8.30–9.10)	8.60 (8.00–9.10)	0.062
Hematocrit, %	35.77 ± 6.58	36.15 ± 6.44	34.56 ± 6.88	<0.01
White blood cells, K/uL	10.90 (8.30–14.00)	10.70 (8.10–13.80)	11.80 (9.10–15.85)	0.003
Red blood cells, m/uL	3.94 ± 0.799	3.99 ± 0.78	3.78 ± 0.85	<0.01
Platelet count, K/uL	201.00 (148.00–262.00)	204.00 (151.00–265.00)	194.00 (129.50–255.50)	0.069
Hemoglobin, g/dL	12.00 (10.30–13.40)	12.20 (10.60–13.50)	11.40 (9.65–12.90)	<0.001
Gender				0.068
Female	393.00 (43.00%)	288.00 (41.26%)	105.00 (48.61%)	
Male	521.00 (57.00%)	410.00 (58.74%)	111.00 (51.39%)	
Race, *n* (%)				0.010
White	487.00 (53.28%)	391.00 (56.02%)	96.00 (44.44%)	
Black	102.00 (11.16%)	77.00 (11.03%)	25.00 (11.57%)	
Asian	30.00 (3.28%)	24.00 (3.44%)	6.00 (2.78%)	
Others/Unknown	295.00 (32.28%)	206.00 (29.51%)	89.00 (41.20%)	
Diabetes, *n* (%)				0.101
No	696.00 (76.15%)	541.00 (77.51%)	155.00 (71.76%)	
Yes	218.00 (23.85%)	157.00 (22.49%)	61.00 (28.24%)	
Pneumonia, *n* (%)				0.209
No	618.00 (67.61%)	480.00 (68.77%)	138.00 (63.89%)	
Yes	296.00 (32.39%)	218.00 (31.23%)	78.00 (36.11%)	
Ischemic heart disease, *n* (%)				0.921
No	711.00 (77.79%)	544.00 (77.94%)	167.00 (77.31%)	
Yes	203.00 (22.21%)	154.00 (22.06%)	49.00 (22.69%)	
Chronic kidney disease, *n* (%)				0.507
No	797.00 (87.20%)	612.00 (87.68%)	185.00 (85.65%)	
Yes	117.00 (12.80%)	86.00 (12.32%)	31.00 (14.35%)	
Hepatic disease, *n* (%)				0.260
No	881.00 (96.39%)	676.00 (96.85%)	205.00 (94.91%)	
Yes	33.00 (3.61%)	22.00 (3.15%)	11.00 (5.09%)	
Hyperlipidemia, *n* (%)				0.695
No	592.00 (64.77%)	455.00 (65.19%)	137.00 (63.43%)	
Yes	322.00 (35.23%)	243.00 (34.81%)	79.00 (36.57%)	
Cancer, *n* (%)				0.799
No	798.00 (87.31%)	611.00 (87.54%)	187.00 (86.57%)	
Yes	116.00 (12.69%)	87.00 (12.46%)	29.00 (13.43%)	
Chronic bronchitis, *n* (%)				0.562
No	868.00 (94.97%)	665.00 (95.27%)	203.00 (93.98%)	
Yes	46.00 (5.03%)	33.00 (4.73%)	13.00 (6.02%)	
Heart failure, *n* (%)				0.185
No	765.00 (83.70%)	591.00 (84.67%)	174.00 (80.56%)	
Yes	149.00 (16.30%)	107.00 (15.33%)	42.00 (19.44%)	
Hypertension, *n* (%)				0.843
No	403.00 (44.09%)	306.00 (43.84%)	97.00 (44.91%)	
Yes	511.00 (55.91%)	392.00 (56.16%)	119.00 (55.09%)	

GCS, Glasgow Coma Scale; RR, respiratory rate; HR, heart rate; Sabp, systolic average blood pressure; Dabp, diastolic average blood pressure; AST, aspartate transaminase; ALT, alanine transaminase; Sapsii, simplified acute physiology score II; Oasis, Oxford acute severity of illness score; PT, prothrombin time; PTT, activated partial thromboplastin time; INR, international normalized ratio.

### Serum bilirubin levels correlate with 28-day all-cause mortality in patients with ICH

**Figure 2:**
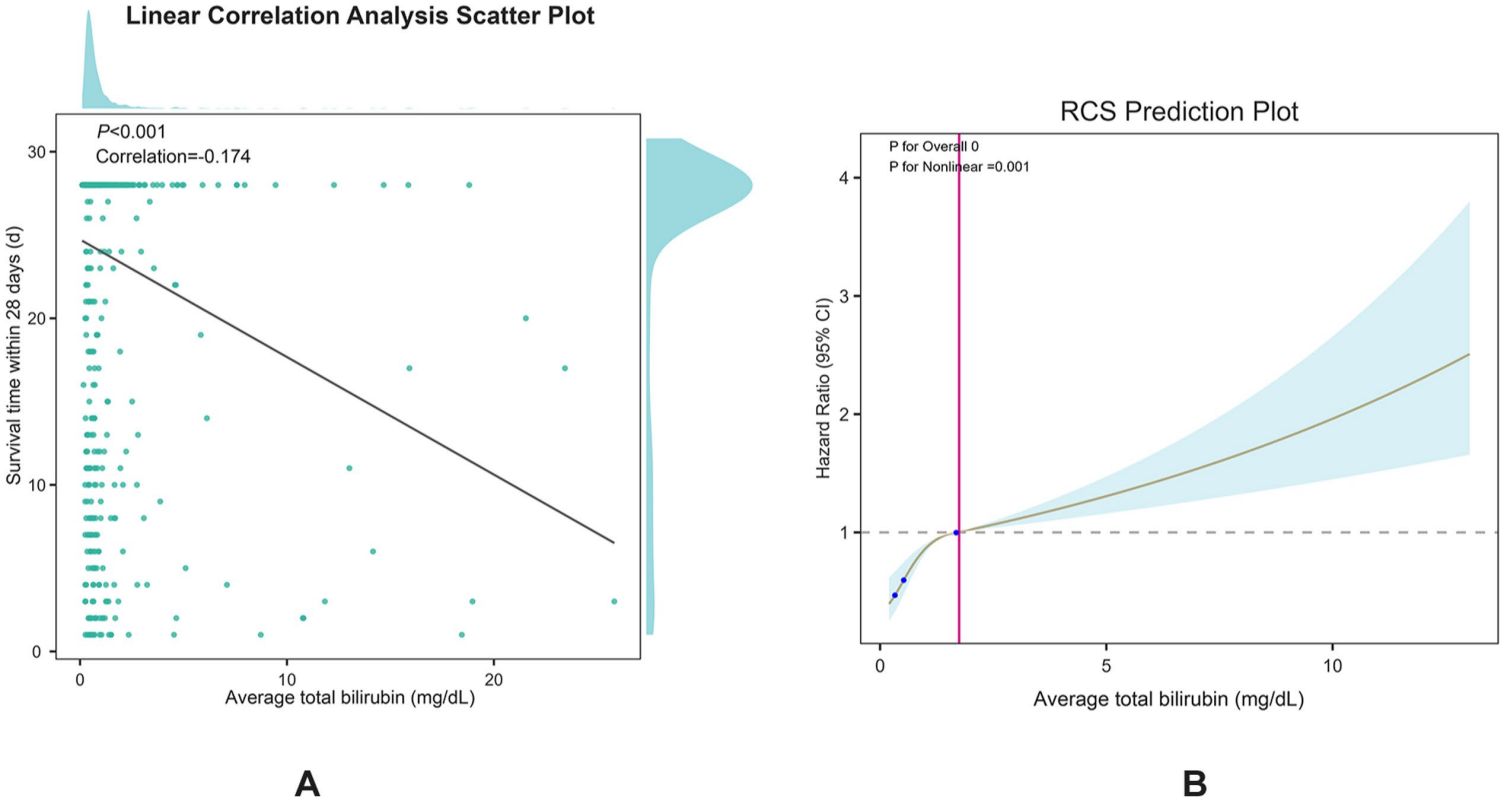
**(A)** spearman correlation test between serum TBIL and 28-day survival time; **(B)** Restricted cubic spline plots for serum TBIL and 28-day all-cause mortality.

**Figure 3:**
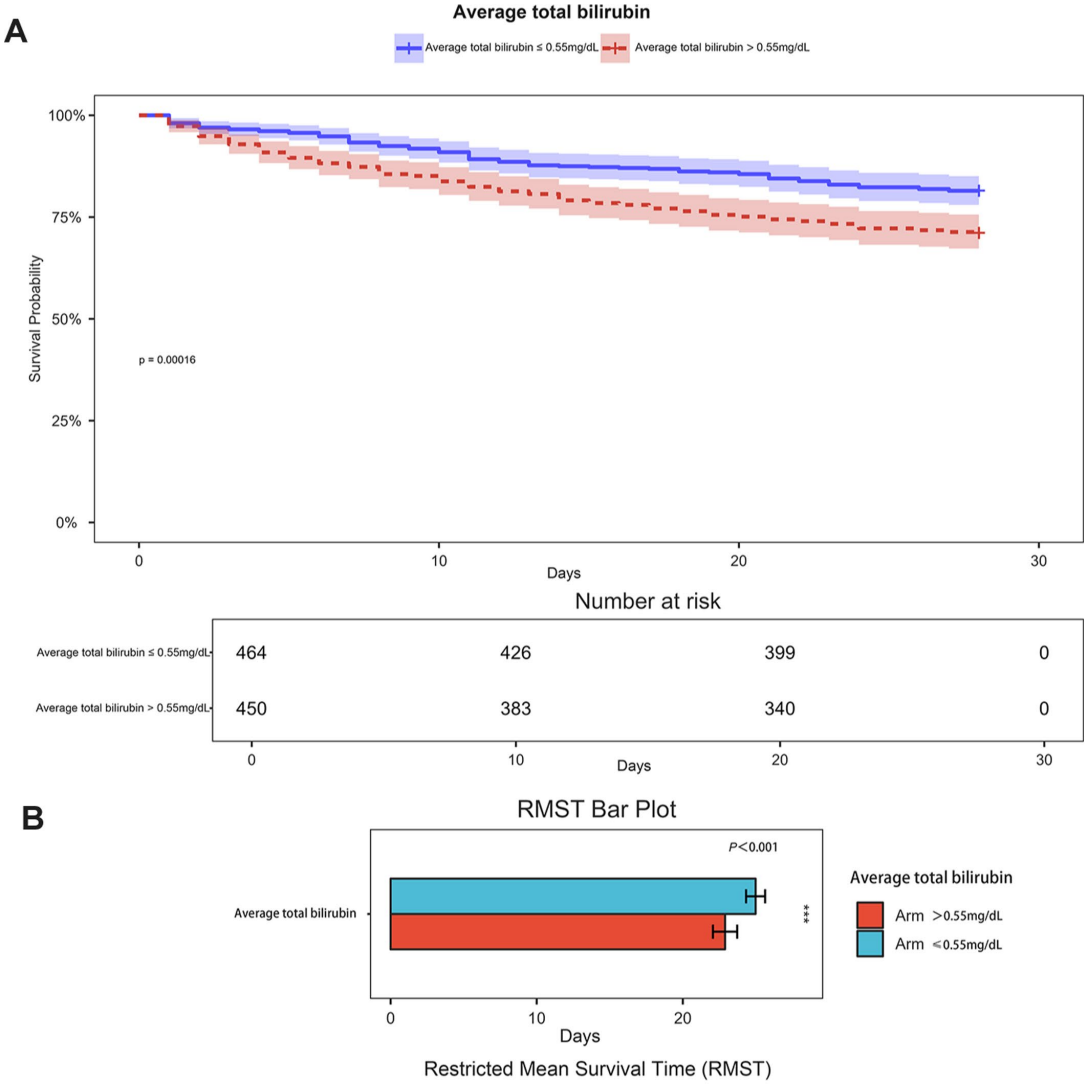
**(A)** Kaplan–Meier curves curve of two groups of patients(grouped by serum TBIL 0.55 mg/dL cutoff); **(B)** Restricted mean survival time bar plot of two groups of patients (grouped by serum TBIL 0.55 mg/dL cutoff).

Spearman’s correlation analysis (Figure 2A) demonstrated a significant negative correlation between serum bilirubin levels and 28-day survival in patients with ICH (*p* < 0.001, correlation = −0.174). Furthermore, restricted cubic spline curve analysis showed a distinct nonlinear correlation between serum bilirubin levels and 28-day all-cause mortality (p for overall = 0, p for nonlinear = 0.001), as shown in Figure 2B. The patients were categorized into two groups according to the median serum TBIL level of 0.55 mg/dL. Kaplan–Meier survival analysis (Figure 3A) showed that the group with higher serum TBIL levels exhibited a more rapid decline in survival over time compared with that of the group with lower serum TBIL levels (*p* = 0.00016). Restricted mean survival time analysis (Figure 3B) revealed that the group with higher serum bilirubin levels had a significantly reduced mean survival time compared with that of the group with lower serum TBIL levels (*p* < 0.001).

### Serum bilirubin level is a significant risk factor for 28-day all-cause mortality in patients with ICH

**Figure 4:**
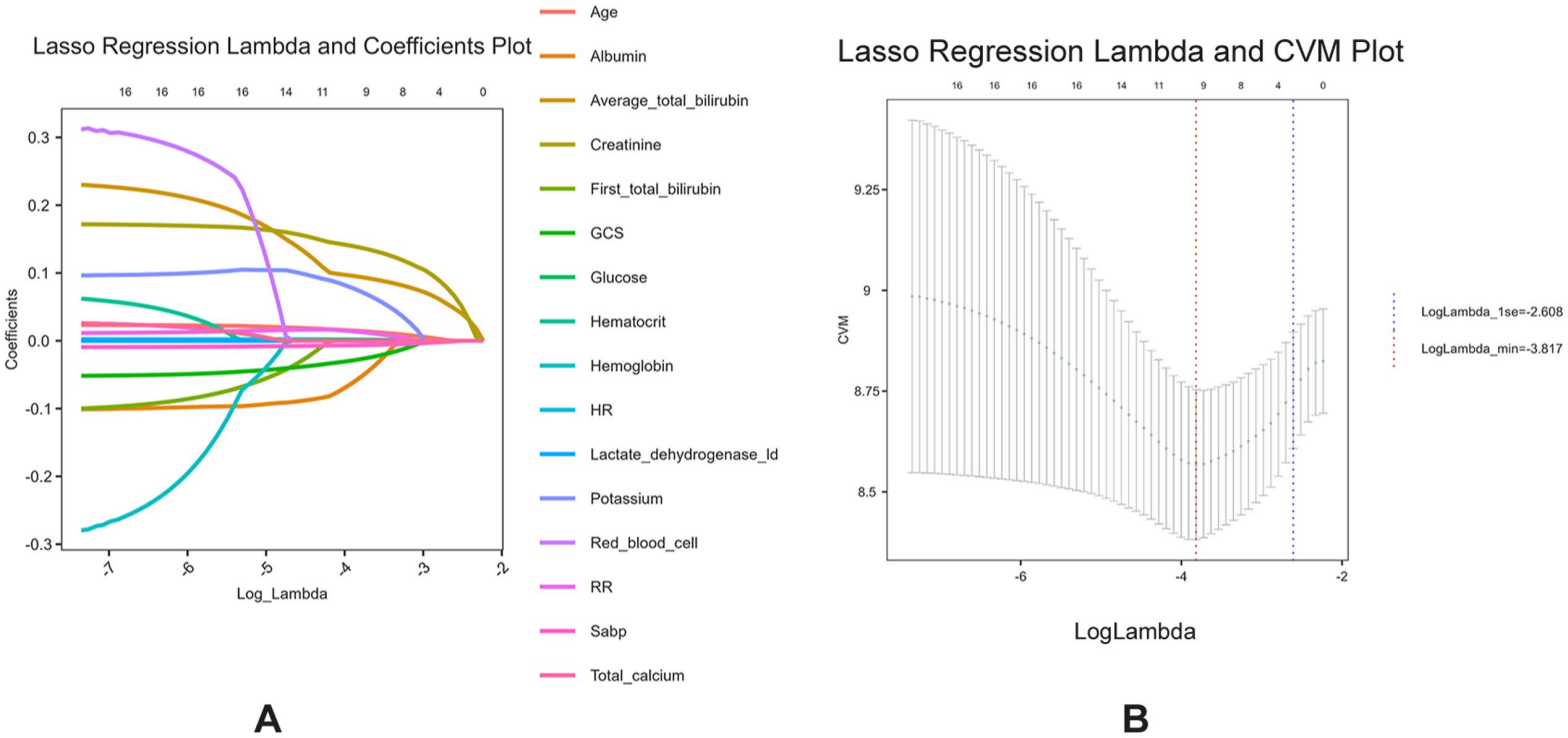
**(A)** Lasso regression lambda value mean square error plot; **(B)** Lasso regression lambda value coefficient plot.

**Figure 5:**
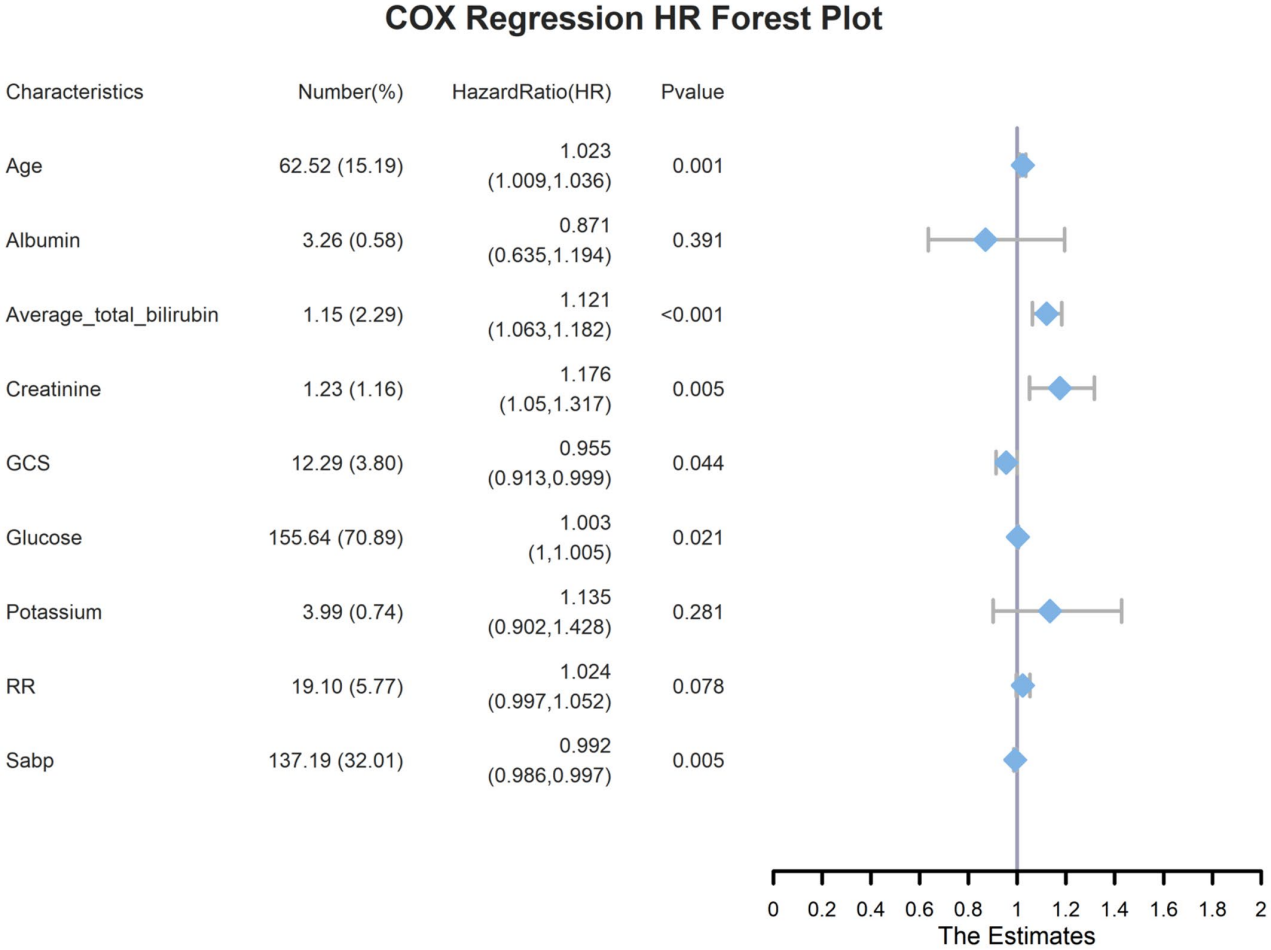
Multivariate COX regression forest plots for the highest weighted influential variables screened by lasso regression.

A preliminary univariate Cox regression analysis identified 15 variables associated with 28-day mortality (Supplementary Table S2), including serum TBIL levels (hazard ratio [95% confidence interval] = 1.114 [1.08–1.149], *p* < 0.001). Subsequently, LASSO regression was performed to filter out possible confounders (Table 2 and Figure 4) and evaluate the influence of these variables on 28-day all-cause mortality. This identified nine variables that had the most significant impact on 28-day all-cause mortality, including average serum TBIL levels (coefficient lambda = 0.0944). These nine variables were further analyzed using multivariate Cox regression (Table 2 and Figure 5). Following adjustment, serum bilirubin level remained a significant risk factor for 28-day mortality in patients with ICH (hazard ratio [95% confidence interval] = 1.121 [1.063–1.182], *p* < 0.001).

**Table 2:** Multivariate COX regression on variables screened by lasso regression.

Lasso regression		Multivariable COX	
Variable	Coeff lamda	HR (95% CI)	*p*-value
Age	0.0145	1.023(1.009–1.036)	0.001
RR	0.0132	1.024(0.997–1.052)	0.078
HR	0		
Sabp	−0.0064	0.992(0.986–0.997)	0.005
GCS	−0.0275	0.955(0.913–0.999)	0.044
Average Total bilirubin	0.0944	1.121(1.063–1.182)	0
Lactate dehydrogenase ld	0		
First total Bilirubin	0		
Potassium	0.0739	1.135(0.902–1.428)	0.281
Creatinine	0.1379	1.176(1.05–1.317)	0.005
Blood glucose	0.0016	1.003(1.00–1.005)	0.021
Albumin	−0.0554	0.871(0.635–1.194)	0.391
Calcium total	0		
Hematocrit	0		
Red blood cells	0		
Hemoglobin	0		

GCS, Glasgow Coma Scale; RR, respiratory rate; HR, heart rate; Sabp, systolic average blood pressure.

### Subgroup analysis

**Figure 6:**
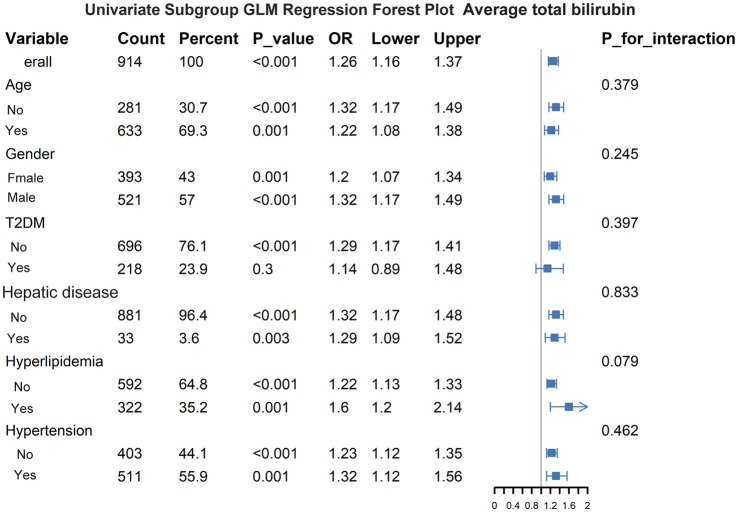
Subgroup analysis of the effect of other variables on the relationship between serum bilirubin and 28-day all-cause mortality.

To further investigate the relationship between serum bilirubin levels and 28-day mortality in patients with ICH, subgroup analyses were conducted (Figure 6). Subgroups were formed based on six factors: age, sex, hypertension, diabetes, hyperlipidemia, and hepatic disease. None of these factors had a significant effect on the association between serum bilirubin levels and 28-day mortality in patients with ICH.

## Discussion

The results of this retrospective study indicate that serum bilirubin levels are associated with 28-day all-cause mortality in patients with ICH. Elevated serum bilirubin levels were inversely correlated with both survival time and rate, with 28 days serving as the study endpoint. High serum bilirubin levels were a significant risk factor for 28-day all-cause mortality.

Serum bilirubin, a byproduct of heme metabolism and breakdown (21), is used as a diagnostic biomarker in clinical settings (22). Evidence indicates that slightly elevated bilirubin levels may protect against certain pathological conditions (23); however, excessively high bilirubin levels can lead to severe encephalopathy (24). Recent research has revealed an increasingly complex relationship between serum bilirubin levels and stroke. Certain studies have suggested that serum bilirubin can diminish stroke risk (25, 26); however, others have linked high bilirubin levels to greater stroke severity and worse outcomes (27, 28). Furthermore, various studies have illustrated the neurotoxic effects of bilirubin in patients with ICH (29–31).

Blood accumulation in the brain parenchyma can enable serum bilirubin to penetrate the brain directly, and additional bilirubin is generated by hemoglobin breakdown within the hematoma. Oxidative processes in the ICH environment can lead to the degradation of bilirubin into oxidized products, which may lead to unfavorable outcomes in patients with ICH (15). However, few studies have assessed the potential of bilirubin as a therapeutic target in the treatment of neurological disorders (9, 22, 32). Our findings suggest that the prognosis of patients with ICH may be improved by reducing serum bilirubin levels.

The association between serum bilirubin levels and the outcomes and prognosis of hemorrhagic stroke is not yet clearly understood. Only one previous study has been performed, a single-center investigation with a small sample size that demonstrated an association of serum bilirubin with ICH severity and prognosis (13). In 276 patients with ICH, higher direct bilirubin levels were associated with increased initial stroke severity and worse clinical outcomes. However, serum TBIL levels did not show a similar pattern, possibly because of the limited number of participants involved. Our study aimed to clarify this issue and discovered a significant negative relationship between serum TBIL levels and 28-day survival, along with a positive correlation with mortality. This finding is consistent with the results of a study that detected higher levels of serum heme oxygenase-1 (HO-1), which converts heme into bilirubin, TBIL, and indirect bilirubin in patients with ICH (33). However, no direct association between the increase in total and indirect bilirubin levels and the increase in HO-1 levels was determined. Based on these findings, we sought to further explore the interrelationships among total, direct, and indirect bilirubin levels in ICH. Unfortunately, more than 90% of the direct and indirect bilirubin level data were missing from the database.

Bilirubin may also have predictive value in patients with ICH. The results of a single-center retrospective study conducted by Jia et al. (17) over an eight-year period suggested that bilirubin could predict hematoma development in patients with ICH. Our findings indirectly indicated that serum bilirubin may predict the outcomes of patients with ICH. As an easily and routinely measured blood component, serum bilirubin is an attractive candidate as a biomarker for the diagnosis and management of ICH.

The typical cutoff value for defining hyperbilirubinemia is 1.2 mg/dL (34). The levels of TBIL levels in the present study were mostly below this value, even in patients who did not survive, which indicates that there was no or little association between ICH mortality and hyperbilirubinemia. This may be due to the fact that, in order to more accurately assess the relationship between ICH and serum TBIL levels, we excluded patients with severe complications related to hepatobiliary system diseases. We regarded serum TBIL levels and hyperbilirubinemia as two distinct entities, and our findings indicate that serum bilirubin level is an independent risk factor for ICH mortality.

This study has some limitations that should be acknowledged. First, the frequency of serum TBIL level measurement varied; some patients were tested only twice, whereas others underwent more than ten assessments. Although we used mean value as a measure, bias was unavoidable. Additionally, the absence of data on direct and indirect bilirubin levels precluded us from exploring the connections between TBIL, direct bilirubin, and indirect bilirubin and the prognosis of patients with ICH. Certain key covariates such as ICH volume, intraventricular hemorrhage, and subventricular location were also absent from the data, which may have introduced bias into the results. Multicenter, large-scale prospective studies are required to address these issues and confirm our findings.

## Conclusion

Higher serum bilirubin levels are associated with a greater risk of mortality within 28 days in individuals with ICH; lower serum bilirubin levels are associated with prolonged survival. Additional studies are necessary to establish a causal relationship between total serum bilirubin levels and outcomes in patients with ICH.

## Data Availability

Publicly available datasets were analyzed in this study. The datasets generated and/or analyzed during the current study are available in the MIMIC-IV datebase (https://physionet.org/content/mimiciv/3.1/).
