# Supplementary material for: Association between serum total bilirubin levels and 28-day all-cause mortality after intracerebral hemorrhage
Source: Front Neurol. 2025 Feb 12;16:1529415. doi: 10.3389/fneur.2025.1529415 (PMC11860087; doi:10.3389/fneur.2025.1529415)
Supplement: Supplementary file 1 [file Table_1.docx]

| **Table S1 Normality tests for key continuous variables** | | | |
| --- | --- | --- | --- |
| VariableName | Kolmogorov_Smirnov_D | Pvalue | Z_Value |
| Age | 0.0480 | 0.0295 | 1.4519 |
| Sapsii | 0.0895 | 0.0000 | 2.7069 |
| Oasis | 0.0703 | 0.0002 | 2.1253 |
| Lactate dehydrogenase ld | 0.3336 | 0 | 10.0856 |
| First bilirubin total | 0.3308 | 0 | 10.0014 |
| Potassium | 0.1051 | 0.0000 | 3.1782 |
| Ast | 0.4423 | 0 | 13.3706 |
| Blood creatinine | 0.2943 | 0 | 8.8986 |
| Blood glucose | 0.1811 | 0 | 5.4747 |
| Blood triglycerides | 0.2957 | 0 | 8.9390 |
| White blood cells | 0.2650 | 0 | 8.0101 |
| Blood sodium | 0.0949 | 0.0000 | 2.8700 |
| Albumin | 0.1069 | 0.0000 | 3.2318 |
| Blood Calcium total | 0.0929 | 0.0000 | 2.8073 |
| ALT | 0.4311 | 0 | 13.0324 |
| Hematocrit | 0.0340 | 0.2415 | 1.0276 |
| Red blood cells | 0.0312 | 0.3352 | 0.9437 |
| Platelet count | 0.0576 | 0.0046 | 1.7419 |
| Hemoglobin | 0.0456 | 0.0445 | 1.3795 |
| Average total bilirubin | 0.3393 | 0 | 10.2582 |
| Dabp | 0.1126 | 0.0000 | 2.3677 |
| Sabps | 0.0719 | 0.0207 | 1.5119 |
| RR | 0.1007 | 0.0000 | 3.0446 |
| HR | 0.0488 | 0.0257 | 1.4757 |
| GCS | 0.229 | 0 | 6.938 |

**Abbreviations:GCS**, Glasgow Coma Scale; **RR**, respiratory rate; **HR**, heart rate;

**Sabp**, systolic average blood pressure; **Dabp**, diastolic average blood

pressure;**AST**,aspartate transaminase;**ALT**,alanine transaminase;**Sapsii**,simplified

acute physiology score II;**Oasis**,Oxford acute severity of illness score.
