# Supplementary material for: Association between serum total bilirubin levels and 28-day all-cause mortality after intracerebral hemorrhage
Source: Front Neurol. 2025 Feb 12;16:1529415. doi: 10.3389/fneur.2025.1529415 (PMC11860087; doi:10.3389/fneur.2025.1529415)
Supplement: Supplementary file 2 [file Table_2.docx]

| **Table S2** **Preliminary screening of ICH 28-day risk factors for all-cause mortality by Univariate cox regression** | | | | |
| --- | --- | --- | --- | --- |
| Characteristics | HazardRatio(HR) | lower_95 | upper_95 | Pvalue |
| Age | 1.021 | 1.012 | 1.031 | 0 |
| HR | 1.008 | 1.001 | 1.015 | \| 0.035 \| \| --- \| |
| RR | 1.025 | 1.003 | 1.048 | 0.001 |
| Sabp | 0.989 | 0.983 | 0.995 | 0 |
| Dabp | 1 | 0.996 | 1.004 | 0.974 |
| Weight | 0.996 | 0.989 | 1.002 | 0.166 |
| Average_bilirubinbilirubin | 1.114 | 1.08 | 1.149 | 0 |
| First total bilirubin | 1.1 | 1.064 | 1.138 | 0 |
| ALT | 1 | 1 | 1 | 0.853 |
| Albumin | 0.722 | 0.579 | 0.899 | 0.004 |
| AST | 1 | 1 | 1 | 0.313 |
| Total Calcium | 0.847 | 0.726 | 0.989 | 0.036 |
| Creatinine | 1.061 | 1.016 | 1.107 | 0.008 |
| Glucose | 1.002 | 1.001 | 1.003 | 0 |
| Hematocrit | 0.97 | 0.951 | 0.989 | 0.002 |
| Hemoglobin | 0.893 | 0.844 | 0.946 | 0 |
| Lactate dehydrogenase ld | 1 | 1 | 1 | 0.008 |
| Platelet count | 0.999 | 0.997 | 1 | 0.115 |
| Potassium | 1.2 | 1.015 | 1.419 | 0.033 |
| Red blood cells | 0.753 | 0.638 | 0.888 | 0.001 |
| Sodium | 1.007 | 0.982 | 1.033 | 0.579 |
| Triglycerides | 1 | 1 | 1.001 | 0.353 |
| White blood cells | 1.004 | 0.998 | 1.009 | 0.197 |
| GCS | 0.922 | 0.889 | 0.957 | 0 |
| gender |  |  |  |  |
| Female |  |  |  |  |
| Male | 0.766 | 0.587 | 1.001 | 0.051 |
| Hepatic |  |  |  |  |
| No |  |  |  |  |
| Yes | 1.519 | 0.828 | 2.787 | 0.177 |
| Heart failure |  |  |  |  |
| No |  |  |  |  |
| Yes | 1.276 | 0.911 | 1.788 | 0.156 |
| Hyperlipidemia |  |  |  |  |
| No |  |  |  |  |
| Yes | 1.051 | 0.797 | 1.386 | 0.724 |
| Hypertension |  |  |  |  |
| No |  |  |  |  |
| Yes | 0.96 | 0.734 | 1.255 | 0.763 |
| Ischemic heart disease |  |  |  |  |
| No |  |  |  |  |
| Yes | 1.037 | 0.754 | 1.426 | 0.822 |
| Pneumonia |  |  |  |  |
| No |  |  |  |  |
| Yes | 1.156 | 0.876 | 1.526 | 0.306 |
| Race |  |  |  |  |
| White |  |  |  |  |
| Black | \| 1.258 \| \| --- \| | \| 0.81 \| \| --- \| | \| 1.953 \| \| --- \| | \| 0.307 \| \| --- \| |
| Asian | 1.02 | \| 0.447 \| \| --- \| | \| 2.328 \| \| --- \| | \| 0.962 \| \| --- \| |
| Others/Unkown | \| 1.64 \| \| --- \| | \| 1.229 \| \| --- \| | \| 2.189 \| \| --- \| | \| 0.001 \| \| --- \| |
| Diabetes |  |  |  |  |
| No |  |  |  |  |
| Yes | 1.294 | 0.962 | 1.741 | 0.088 |
| Cancer |  |  |  |  |
| No |  |  |  |  |
| Yes | 1.038 | 0.702 | 1.535 | 0.851 |
| Chronic bronchitis |  |  |  |  |
| No |  |  |  |  |
| Yes | 1.243 | 0.709 | 2.177 | 0.448 |
| Chronic kidney |  |  |  |  |
| No |  |  |  |  |
| Yes | 1.15 | 0.786 | 1.682 | 0.471 |
| **Abbreviations:GCS**, Glasgow Coma Scale; **RR**, respiratory rate; **HR**, heart rate;  **Sabp**, systolic average blood pressure; **Dabp**, diastolic average blood  pressure;**AST**,aspartate transaminase;**ALT**,alanine transaminase;**Sapsii**,simplified  acute physiology score II;**Oasis**,Oxford acute severity of illness score | | | | |
